# Controlling the on/off current ratio of ferroelectric field-effect transistors

**DOI:** 10.1038/srep12094

**Published:** 2015-07-10

**Authors:** Ilias Katsouras, Dong Zhao, Mark-Jan Spijkman, Mengyuan Li, Paul W. M. Blom, Dago M. de Leeuw, Kamal Asadi

**Affiliations:** 1 Max-Planck Institute for Polymer Research, Ackermannweg 10, 55128, Mainz, Germany; 2 Holst Centre, High Tech Campus 31, 5656AE, Eindhoven, The Netherlands; 3 ASML, De Run 6501, 5504 DR, Veldhoven, The Netherlands; 4 China Photovoltaic Industry Association, No. 27 Wanshou Road, 100846, Beijing, China

## Abstract

The on/off current ratio in organic ferroelectric field-effect transistors (FeFETs) is largely determined by the position of the threshold voltage, the value of which can show large device-to-device variations. Here we show that by employing a dual-gate layout for the FeFET, we can gain full control over the on/off ratio. In the resulting dual-gate FeFET the ferroelectric gate provides the memory functionality and the second, non-ferroelectric, control gate is advantageously used to set the threshold voltage. The on/off ratio can thus be maximized at the readout bias. The operation is explained by the quantitative analysis of charge transport in a dual-gate FeFET.

Ferroelectric materials have emerged in microelectronics as attractive candidates for data storage applications, *i.e.* non-volatile memory elements that retain their data when the power is turned off, and that furthermore can be read, programmed and erased electrically^1^,^2^,^3^,^4^,^5^. In particular, interest in organic ferroelectric materials^6^,^7^,^8^ is growing due to their low processing temperature that allows device fabrication on foils, enabling thereby flexible electronics^9^,^10^,^11^,^12^. The most commonly used organic ferroelectric materials are poly(vinylidenefluoride) (PVDF) and its random copolymers with trifluoroethylene (P(VDF-TrFE))^13^,^14^,^15^. A non-volatile memory element is the ferroelectric field-effect transistor (FeFET), where the ferroelectric material is used as the gate dielectric^16^. Solution-processed organic FeFETs based on PVDF and P(VDF-TrFE) in combination with various semiconducting polymers and metal-oxides have been reported^17^,^18^,^19^,^20^. A reconfigurable memory array of 250 kbit has been demonstrated^21^.

The memory functionality is obtained by polarizing the ferroelectric gate, which modulates the charge carrier density in the semiconductor channel at the semiconductor/ferroelectric interface. Presence of compensation charges in the semiconductor is crucial for the operation of the FeFET. The two ferroelectric polarization states of the gate dielectric can only be stabilized when holes or electrons can be accumulated in the channel. Absence of these compensation charges leads to depolarization of the ferroelectric gate. Depending on the polarization state, the FeFET is programmed into a high conductive on-state or a low conductive off-state. The ratio of the on- and off-state currents, probed at zero gate bias at a low drain bias, is defined as the on/off ratio. Integration into large memory arrays requires FeFETs with well-defined on/off ratio.

In Fig. 1 a schematic of a typical transfer characteristic for a FeFET based on a *p*-type organic semiconductor is given in red. The threshold voltage, *V*_*th1*_, is slightly positive. Hence, at zero gate bias there is current flowing between the source and drain electrodes. When the negative gate bias exceeds the coercive voltage, *-V*_*c*_, the ferroelectric gate polarizes. Since the semiconductor is *p*-type, it can supply holes for compensation and stabilization of the negative polarization charges. As the ferroelectric polarization is much larger than the dielectric charge density, the current shows an abrupt increase at the coercive voltage. The transistor is in the on-state.

Upon scanning back the gate voltage to zero bias, the ferroelectric gate remains polarized and the current remains high. Upon further increase of the gate bias, the polarization remains stable until the positive coercive voltage, *+V*_*c*_, is exceeded. The polarization of the ferroelectric gate then switches direction and positive surface charges accumulate. Since the semiconductor is *p*-type, it cannot provide electrons as compensating charges. Hence the ferroelectric polarization is unstable and the gate depolarizes^22^. As a consequence the current drops and the transistor switches to the off-state. In unipolar *p*-type ferroelectric transistors therefore, the ferroelectric is either negatively polarized in the on-state or depolarized in the off-state.

The current in the on-state is dominated by the ferroelectric polarization. Hence the on-state current at zero gate bias does not depend on the threshold voltage. In contrast, the off-state current strongly depends on the value of the threshold voltage. As shown in Fig. 1, the threshold voltage of the organic FeFET determines the maximum attainable on/off ratio. To achieve a high on/off ratio in a *p*-type FeFET, the threshold voltage should be negative. In organic field-effect transistors however, the threshold voltage is typically positive and the transistor is normally-on^23^,^24^. The value of the threshold voltage can show large device-to-device variations. In addition, the threshold voltage is influenced by the environment and by the duty cycle history^25^. As a result, the on/off current ratio of discrete FeFETs can show a large parameter spread.

Here we address the issue of threshold voltage spread in FeFETs. By introducing a second gate layer we gain full control over the on/off ratio. The resulting dual-gate transistor has been suggested for information storage^26^,^27^,^28^ Here we show that the dual-gate FeFET allows setting the threshold voltage of the transistor at the desired value by independently varying the voltages on both gates^29^,^30^. The ferroelectric gate provides the memory functionality and the second, non-ferroelectric, control gate is advantageously used to set the threshold voltage. In the resulting dual-gate FeFET the on/off ratio is optimized by the control gate. We explain the operation of a dual-gate FeFET by a quantitative analysis of the charge transport.

## Results and Discussion

### FeFET characterization

**Figure 2:**
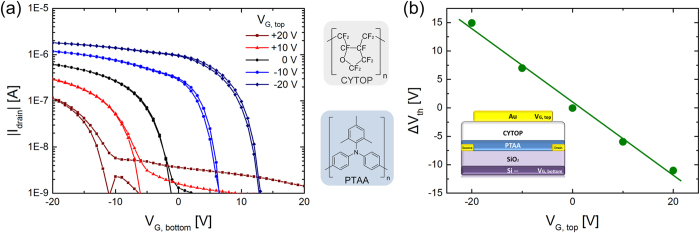
Threshold voltage control in a dual-gate FET. **(a)** Transfer curves of a polytriarylamine dual-gate field-effect transistor. The channel length and width are 10 μm and 10000 μm respectively. The absolute value of the drain current is presented on a semi-logarithmic scale as a function of the bottom gate bias. The top gate bias is varied from +20 V to −20 V, in 10 V steps. **(b)** The threshold voltage depends on the top gate bias as *Δ*V_th_ = −C_top_/C_bottom_ ×V_G, top_, where C_top_ and C_bottom_ are the top and bottom gate capacitances. The inset shows the device layout. The chemical structures of the semiconductor and the top gate dielectric are indicated.

As a first step, we characterized the performance of a conventional non-ferroelectric dual-gate FET. The transfer curves were obtained by scanning the voltage on the bottom gate for various fixed top gate biases. The voltages on both gates were varied independently. The measurements are illustrated in Fig. 2a. At zero top gate bias, the device behaves as a regular field-effect transistor. The saturated source-drain current is about 1 μA and the threshold voltage is about zero volt. Figure 2a shows that the threshold voltage shifts systematically with the applied top gate bias.

The threshold voltage shift,*ΔV*_*th*_, can be quantified from the total charge, *Q*_*total*_, induced by the two gates:


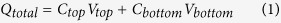


where *C*_*top*_ and *C*_*bottom*_ are the top and bottom dielectric capacitances per unit area. At the threshold voltage the total induced charge is zero. If the top gate is fixed and the bottom gate is swept, then the shift in threshold voltage is given by:


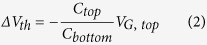


where *V*_*G,top*_ is the fixed top gate bias. Figure 2b shows the relationship between experimental shift in threshold voltage, *ΔV*_*th*_, and *V*_*G,top*_. The dependence is linear and well described by Eq. (2).

**Figure 3:**
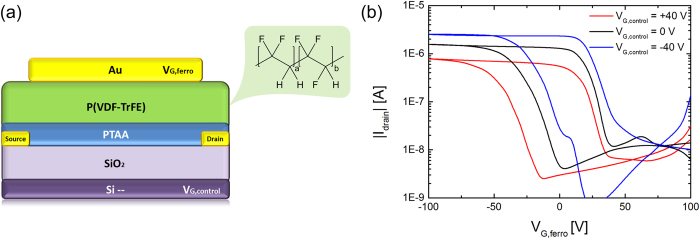
Dual-gate FeFET. **(a)** Layout of the dual-gate FeFET. The chemical structure of the ferroelectric material is indicated. **(b)** Transfer curves of a dual-gate FeFET. The source-drain current is measured upon scanning the ferroelectric top gate. The transfer curves are presented for different fixed biases on the control, bottom gate. The channel length and width are 10 μm and 10000 μm respectively. The black line shows the FeFET at zero bias on the control gate. The red and blue lines represent the transfer curve for a fixed control gate bias of +40 V and −40 V, respectively.

Next, we characterized the performance of a dual-gate FeFET. The device layout is schematically presented in Fig. 3a. The drain bias was fixed at −20 V and the gate bias on the ferroelectric top gate was swept from −100 V to +100 V and back. The relatively high top gate bias is due to the thickness of the ferroelectric gate dielectric, which was not optimized. The high gate voltage, however, does not affect the operation mechanism. The source electrode was grounded. The transfer curves for three different control gate biases of 0 V and ±40 V are presented in Fig. 3b. First we characterize the bottom channel. We applied a zero volt top gate bias. The measured source-drain current is similar to that measured in Fig. 2a at the same bias. To characterize the top channel, we applied a zero bottom gate bias. We note that the same current is measured when using a floating bottom gate. The electrical transport can qualitatively be understood as follows. The ferroelectric switches when the coercive voltage is applied to the ferroelectric top gate, irrespective of the bias on the SiO_2_ control gate. The on-current at 0 V top gate bias is dominated by the ferroelectric polarization. Hence in first order approximation it is expected that the on-current does not depend on the control gate bias. In practice however, the on-current does depend on the control, bottom gate bias. The origin is partial channel depletion. The main effect of the additional control gate is a shift of the threshold voltage, as described by Eq. (2). Hence by adjusting the control gate bias, the on/off current ratio can be tuned. A phenomenological description of the transfer curves is given below. A quantitative analysis is presented in the next section.

The FeFET was characterized by applying 0 V on the SiO_2_ control gate. Typical transfer curves for *p*-type FeFETs were obtained, as shown by the black line in Fig. 3b. When the top gate bias is at −100 V, the ferroelectric is negatively polarized and the polarization is stable after removal of the gate bias due to accumulation of holes in the PTAA channel. As the gate voltage increases towards positive values, the drain current remains unchanged until about +10 V, where the polarization of the ferroelectric starts to switch direction. The channel is depleted from holes and the drain current drops. The positive polarization of the ferroelectric is not stable due to the lack of electrons in the semiconductor channel. For the back sweep, from positive to negative gate bias, the channel is depleted at the beginning; the current remains low until the threshold voltage. As the gate bias becomes lower than the threshold voltage the transistor enters the hole accumulation regime. A conducting channel is formed and current flows between the source and drain electrodes. At larger negative gate biases the ferroelectric switches and at about −70 V it is again fully polarized. We note that device optimization towards a low-voltage operation is an art in itself and beyond the scope of the present paper. Here we demonstrate the proof of concept and we discuss the device physics.

In the next step, the control gate bias is set at +40 V. The transfer curve of the FeFET is presented by the red curve in Fig. 3b. Application of a positive bias on the control SiO_2_ gate has two effects on the FeFET performance. Firstly, as compared to the case of zero bias on the control gate, the threshold voltage of the FeFET is shifted to the left, towards a negative value of about −20 V. Hence the off-current at 0 V gate bias decreases. Secondly, the drain current in the on-state is lower. Lowering of the on-state drain current is due to the partial depletion of the conductive channel due to the positive bias on the SiO_2_ control gate. Since PTAA is a *p*-type semiconductor, it cannot supply electrons. A positive control gate bias cannot be compensated. The control gate field is therefore unscreened and penetrates through the semiconductor layer. As a result, the top channel is electrostatically partially depleted and therefore the current is lower. Despite the lower on-state current the on/off ratio remains constant due to the lowering of the off-state current.

Next we apply −40 V on the control SiO_2_ gate. The transfer curve is shown as the blue line in Fig. 3b. Application of a negative bias on the control SiO_2_ gate has two effects on the FeFET performance. Firstly, as compared to the case of zero bias on the control gate, the threshold voltage of the FeFET is shifted to the right, towards a positive value of about +20 V. Hence the off-current at 0 V gate bias increases. Secondly, the drain current in the on-state is slightly higher due to the formation of an additional conductive channel in PTAA at the SiO_2_ interface. The negative bias on the control gate accumulates holes in PTAA at the SiO_2_ interface, which contribute to the total device current. Despite the increase in the on-state current, the on/off ratio of the FeFET is significantly decreased.

**Figure 4:**
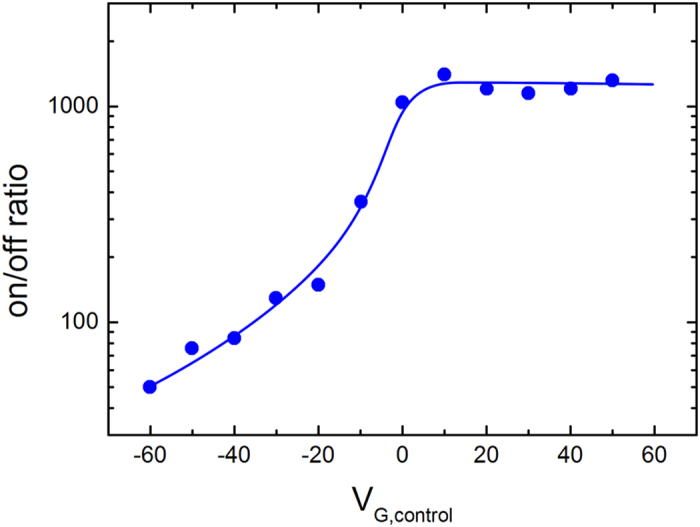
On/off current ratio of a dual-gate FeFET. The on/off current ratio is plotted as a function of the bias on the control gate. The on/off ratio is large for positive control gate bias and decreases with negative control gate bias. At control gate voltages larger than +10 V the bottom channel is fully depleted. As a consequence the on/off ratio saturates. The solid line is a guide to the eye.

We show that the on/off current ratio is sensitive to the bias applied to the control gate. The ferroelectric gate was programmed in the on- and off-state and subsequently grounded. The corresponding state currents were recorded as a function of bias on the control gate. The on/off current ratio as a function of the bias on the control gate is presented in Fig. 4. For negative biases applied on SiO_2_ control gate the on/off ratio is drastically reduced. In contrast, for positive SiO_2_ gate biases the on/off ratio is maximized. The origin is tuning of the threshold voltage by the control gate, as described above. The large range of control gate biases at which the on/off ratio is maximum and constant makes the transistor robust to spurious external signals.

**Figure 5:**
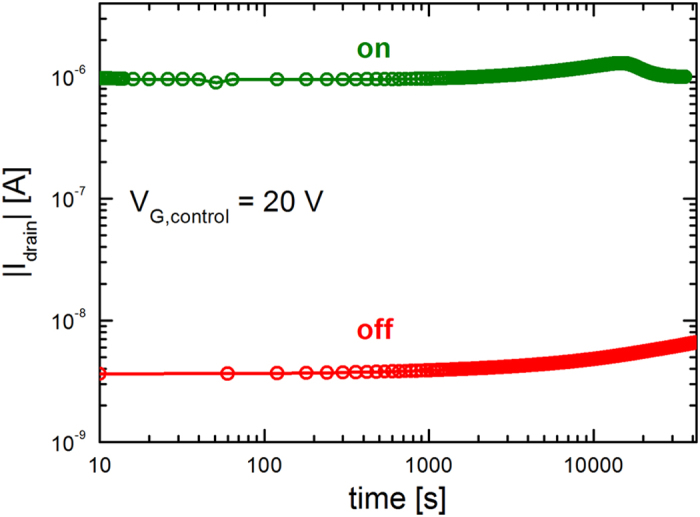
Data retention time of dual-gate FeFET. The ferroelectric gate was programmed either in the on- or off-state and subsequently grounded. The drain current was measured using a drain bias of −20 V and a control gate bias of +20 V. The on- and off-state currents are presented as a function of time. The on/off ratio is stable up to 40000 seconds, i.e. more than 10 hours.

The retention of the polarization was measured to ensure that there are no adverse effects on the FeFET performance due to *e.g.* stress^25^. The ferroelectric gate was programmed either in the on- or off-state and subsequently grounded. The drain current was measured using a drain bias of −20 V and a control gate bias of +20 V. The corresponding currents as a function of time are presented in Fig. 5. The on/off ratio is constant for more than 10 hours, in good agreement with retention measurements on conventional FeFETs^27^,^31^.

We note that the channel conductance of a FeFET is controlled by switching at the source electrode^32^. Therefore the application of the control gate bias does not influence the switching time.

### Device physics of dual-gate FeFETs

Here we provide a quantitative analysis of the charge transport in a dual-gate FeFET. The operation is related to the capacitances of the gate dielectrics and of the semiconductor layer. Assuming a parallel plate capacitor, we calculated *C*_*ferro*_ = 8.85 nF/cm^2^ for the P(VDF-TrFE) layer (*ε* = 10), *C*_*semi*_ = 53 nF/cm^2^ for the PTAA semiconductor layer (*ε* = 3) and *C*_*control*_ = 3.5 nF/cm^2^ for the SiO_2_ layer (*ε* = 3.9). We first consider the case of a fixed negative bias on the bottom, control gate. An accumulation layer is formed at the control gate dielectric/semiconductor interface. Starting with a depolarized ferroelectric gate, the device is simply a conventional dual-gate transistor. The threshold voltage shift is given by:


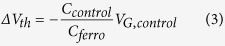


comparable to Eq. (2). At a *V*_*G, control*_ of −40 V, the threshold voltage shift of the dual gate FeFET transistor is calculated as about 16 V, in good agreement with the experimentally observed value of about 15 V, as seen in Fig. 3b. Therefore, at a *V*_*G, ferro*_ of 0 V the off-state current of the dual-gate FeFET is increased.

On the other hand, when the ferroelectric gate is polarized in the on-state, a second accumulation layer is formed at the ferroelectric/semiconductor interface. The ferroelectric gate remains fully polarized at a *V*_*G, ferro*_ of 0 *V.* The control gate field is fully screened by the bottom accumulation channel. Both channels are independent. The total on-state current of the dual-gate FeFET is the sum of the currents flowing in each channel. Despite this slightly higher current in the on-state, the on/off ratio of the dual-gate FeFET is lowered due to the increased off-state current.

We now consider the case when the control gate is kept at fixed positive bias. Since PTAA is a *p*-type semiconductor, it cannot supply electrons. Thus when a positive gate bias is applied on the control gate, the bottom-channel is depleted. Starting with the depolarized off-state of the ferroelectric top-gate, the device is again a dual gate transistor. The depleted part of the semiconductor forms a capacitor in series with the control gate dielectric. Then, in Eq. (3) *C*_*control*_ has to be replaced by:


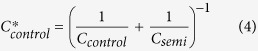


The threshold voltage shift at a *V*_*G, control*_ of +40 V, is then calculated to be about −15 V, in good agreement with the experimentally observed value of about −20 V, as shown in Fig. 3. The threshold voltage of the dual-gate FeFET is now negative, which implies that at a *V*_*G, ferro*_ of 0 *V*, the off-state current that is running between the electrodes is only the leakage current, here 1–2 nA.

We now calculate the current in the on-state of the dual-gate FeFET. Due to the positive gate bias on the control gate the bottom-channel is depleted. Therefore the bottom-gate field is unscreened and penetrates through the semiconductor layer, electrostatically affecting the top channel. The ferroelectric is fully polarized. The polarization charge is compensated for by charge carriers in the channel and by the unscreened control gate field. The amount of charges in the semiconductor channel can be estimated as follows.

**Figure 6:**
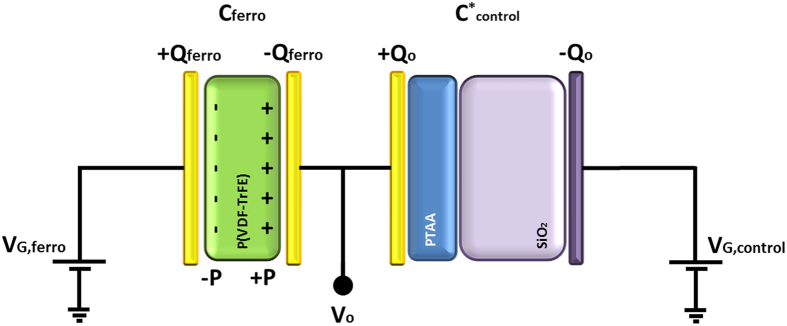
Equivalent circuit for a dual-gate FeFET. The ferroelectric gate dielectric is in series with the depleted semiconductor/control gate dielectric stack. The device layout is shown in Fig. 3a.

In a circuit consisting of a ferroelectric capacitor, *C*_*ferro*_, in series with a linear capacitor, *C*_*s*_, Black *et al.*^33^ have shown that the charge over the ferroelectric capacitor, *Q*_*ferro*_, is equal to:


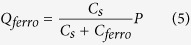


where *P* is the polarization. To describe a dual-gate FeFET, we extend the analysis of Black *et al.* by including the additional control gate. The equivalent circuit is shown in Fig. 6. We assume that the ferroelectric remains fully polarized. The ferroelectric gate, *V*_*G, ferro*_, is grounded. The voltage over the ferroelectric, *V*_*f*_, plus the voltage drop over the depleted semiconductor/control gate dielectric stack, *V*_*o*_, equals the applied control gate bias, *V*_*G, control*_:


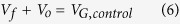


The central node of the circuit is electrically neutral, meaning that the charge at the ferroelectric, *Q*_*ferro*_, equals the charge over the semiconductor/control gate dielectric stack, *Q*_*o*_. Eq. (6) becomes:


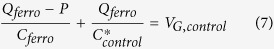


where *Q*_*ferro*_*-P* / *C*_*ferro*_ is the voltage over the ferroelectric and *P* is the remanent polarization. For *Q*_*ferro*_ we then obtain:


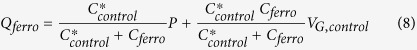


When the control gate is grounded, Eq. (8) reduces to Eq. (5), as derived in reference 33. For a control gate bias of +40 V and a remanent polarization of 7 μC/cm^2^ we obtain a value of 2 μC/cm^2^ for *Q*_*ferro*_.

In the derivation we have treated the semiconductor as a pure insulator. In reality the polarized ferroelectric gate induces a charge density of 7 μC/cm^2^ in the semiconductor channel. The calculated *Q*_*ferro*_ corresponds to the depleted charge density due to the presence of the unscreened control gate. The charge carrier density in the channel amounts to the polarization-induced charge minus *Q*_*ferro*_. For the present case of a control gate bias of +40 V, we calculate a reduction of 30% in the charge carrier density.

The charge carrier density reduction can also be directly calculated from the on-state current. Since the current between source and drain has a linear relationship with charge density, the on-state current therefore should reach 70% of its original value when the control gate is grounded. Fig. 3b shows that the on-state currents are 1.1 × 10^−6^ A and 7.5 × 10^−7^ A for *V*_*G, control*_ of 0 V and +40 V, respectively. The ratio between these currents is 69%, in good agreement with the estimated 70%, as calculated above.

### Summary and Conclusion

In summary, we have investigated the device physics of a dual-gate FeFET. The transistors comprised a ferroelectric gate of P(VDF-TrFE) and an SiO_2_ control gate. The ferroelectric gate provides the memory functionality and the non-ferroelectric, control gate, is used to fine tune the position of the threshold voltage. The on/off ratio could thus be maximized at the readout bias. We related the operation to the capacitances of the gate dielectrics and of the semiconductor layer. The charge transport in a dual-gate FeFET could be quantitatively interpreted.

## Methods

Dual-gate FeFETs were fabricated on heavily doped *n*-type Si monitor wafers that act as a common control gate. The gate dielectric is a 1 μm thermally grown SiO_2_ oxide layer. We note that SiO_2_ can be replaced by organic gate dielectrics, which are a prerequisite for practical applications. However, here we are only interested in the operation mechanism of discrete dual-gate FeFETs. Gold source and drain electrodes, 50 nm thick, were defined by photolithography using a 2 nm Ti adhesion layer. The SiO_2_ layer was passivated with hexamethyldisilazane prior to semiconductor deposition. Polytriarylamine (PTAA) films with a layer thickness of approximately 80 nm were spin-coated from toluene. On the PTAA, the ferroelectric gate dielectric P(VDF-TrFE) (80/20 mol %) was spin-coated from butanone. The P(VDF-TrFE) layer thickness was measured with a Dektak profilometer and amounted to 1 μm. Subsequently the transistor was annealed at 140 °C in vacuum to enhance the crystallinity of the P(VDF-TrFE) layer. The top gate Au electrode was evaporated through a shadow mask. Using the same device fabrication recipe, dual-gate field-effect transistors with CYTOP^TM^ as a non-ferroelectric top gate, were also fabricated. Electrical measurements were performed in vacuum and in ambient conditions using an Agilent HP4155C semiconductor parameter analyser.

## Additional Information

**How to cite this article**: Katsouras, I. *et al.* Controlling the on/off current ratio of ferroelectric field-effect transistors. *Sci. Rep.* **5**, 12094; doi: 10.1038/srep12094 (2015).

## Figures and Tables

**Figure 1:**
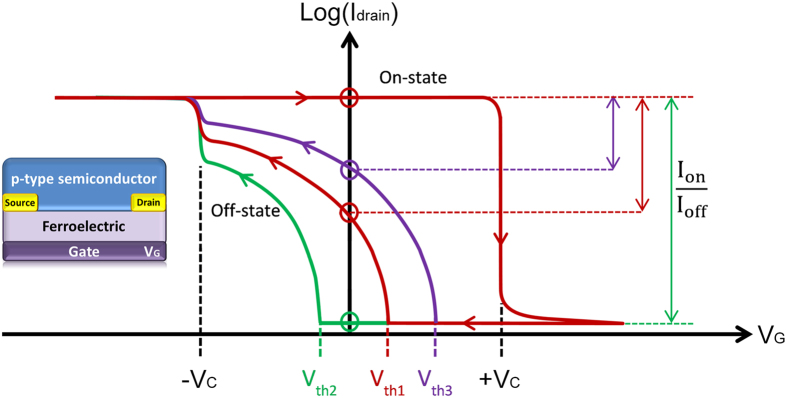
Transfer curves of a unipolar p-type FeFET. Schematic transfer curves are depicted for three different values of the threshold voltage. The memory window for a p-type FeFET is the difference between threshold voltage and positive coercive field. It is shown that the corresponding on/off ratio, defined as the ratio of the on- and off-state currents at zero gate bias, varies with the threshold voltage. The FeFET device layout is indicated.

## References

[b1] Kosman M. S. On the nature of ferroelectricity in Rochelle salt. J. Exp. Theor. Phys. 19, 899–907 (1949).

[b2] Naber R. C. G., Asadi K., Blom P. W. M., De Leeuw D. M. & De Boer B. Organic nonvolatile memory devices based on ferroelectricity. Adv. Mater. 22, 933–945 (2010). 20217816 10.1002/adma.200900759

[b3] Heremans P. *et al.* Polymer and organic nonvolatile memory devices Chem. Mater. 23, 341–358 (2011).

[b4] Ling Q.-D. *et al.* Polymer electronic memories: Materials, devices and mechanisms. Prog. Polym. Sci. 33, 917–978 (2008).

[b5] Ducharme S., Reece T. J., Othon C. M. & Rannow R. K. Ferroelectric polymer Langmuir-Blodgett films for nonvolatile memory applications. IEEE T. Device Mat. Re. 5, 720–735 (2005).

[b6] Cantatore E. *et al.* A 13.56-MHz RFID system based on organic transponders. IEEE J. Solid-St. Circ. 42, 84–92 (2007).

[b7] Roberts M. E. *et al.* Water-stable organic transistors and their application in chemical and biological sensors. P. Natl. Acad. Sci. USA 105, 12134–12139 (2008). 10.1073/pnas.0802105105 PMC2527878 18711145

[b8] Scott J. C. & Bozano L. D. Nonvolatile memory elements based on organic materials. Adv. Mater. 19, 1452–1463 (2007).

[b9] Khan M. A., Bhansali U. S. & Alshareef H. N. High-performance non-volatile organic ferroelectric memory on banknotes. Adv. Mater. 24, 2165–2170 (2012). 22438135 10.1002/adma.201200626

[b10] Sekitani T. *et al.* Printed nonvolatile memory for a sheet-type communication system. IEEE T. Electron Dev. 56, 1027–1035 (2009).

[b11] Ng T. N., Russo B., Krusor B., Kist R. & Arias A. C. Organic inkjet-patterned memory array based on ferroelectric field-effect transistors. Org. Electron. 12, 2012–2018 (2011).

[b12] Furukawa T., Takahashi Y. & Nakajima T. Recent advances in ferroelectric polymer thin films for memory applications. Curr. Appl. Phys. 10, E62–E67 (2010).

[b13] Furukawa T. Ferroelectric properties of vinylidene fluoride copolymers. Phase Transit. 18, 143–211 (1989).

[b14] Nalwa H. S. Ferroelectric Polymers: Chemistry, Physics, and Applications (Marcel Dekker, New York, 1995).

[b15] Kepler R. G. & Anderson R. A. Ferroelectric polymers. Adv. Phys. 41, 1–57 (1992).

[b16] Naber R. C. G. *et al.* High-performance solution-processed polymer ferroelectric field-effect transistors. Nat. Mater. 85, 243–248 (2005).

[b17] Brondijk J. J., Asadi K., Blom P. W. M. & De Leeuw D. M. Physics of organic ferroelectric field-effect transistors. J. Polym. Sci. Pol. Phys. 50, 47–54 (2012).

[b18] Kang S. J. *et al.* Non-volatile ferroelectric poly(vinylidene fluoride-co-trifluoroethylene) memory based on a single-crystalline tri-isopropylsilylethynyl pentacene field-effect transistor. Adv. Funct. Mater. 19, 1609–1616 (2009).

[b19] Yoon S. M. *et al.* Fully transparent non-volatile memory thin-film transistors using an organic ferroelectric and oxide semiconductor below 200 degrees C. Adv. Funct. Mater. 20, 921–926 (2010).

[b20] Park Y. J., Bae I. S., Kang S. J., Chang J. & Park C. Control of thin ferroelectric polymer films for non-volatile memory applications. IEEE T. Dielect. El. In. 17, 1135–1163 (2010).

[b21] Van Breemen A. *et al.* Ferroelectric transistor memory arrays on flexible foils. Org. Electron. 14, 1966–1971 (2013).

[b22] Naber R. C. G. *et al.* Origin of the drain current bistabilitu in polymer ferroelectric field-effect transistors. Appl. Phys. Lett. 90, 113509 (2007).

[b23] Iba S. *et al.* Control of threshold voltage of organic field-effect transistors with double-gate structures. Appl. Phys. Lett. 87, 023509 (2005).

[b24] Horowitz G., Hajlaoui R., Bouchriha H., Bourguiga R. & Hajlaoui M. The concept of “threshold voltage” in organic field-effect transistors. Adv. Mater. 10, 923–927 (1998).

[b25] Bobbert P. A., Sharma A., Mathijssen S. G. J., Kemerink M. & de Leeuw D. M. Operational stability of organic field-effect transistors. Adv. Mater. 24, 1146–1158 (2012). 22298508 10.1002/adma.201104580

[b26] Khan M. A., Caraveo-Frescas J. A. & Alshareef H. N. Hybrid dual gate ferroelectric memory for multilevel information storage. Org. Electron. 16, 9–17 (2015).

[b27] Li M. *et al.* Revisiting the delta-phase of poly(vinylidene fluoride) for solution-processed ferroelectric thin films. Nat. Mater. 12, 433–438 (2013). 23503012 10.1038/nmat3577

[b28] Yoon S. M., *et al.* Oxide semiconductor-based organic/inorganic hybrid dual-gate nonvolatile memory thin-film transistor. IEEE T. Electron Dev. 58, 2135–2142 (2011).

[b29] Spijkman M.-J. *et al.* Dual-gate organic field-effect transistors as potentiometric sensors in aqueous solution. Adv. Funct. Mater. 20, 898–905 (2010).

[b30] Spijkman M.-J. *et al.* Dual-gate thin-film transistors, integrated circuits and sensors. Adv. Mater. 23, 3231–3242 (2011). 21671446 10.1002/adma.201101493

[b31] Naber R. C. G., De Boer B., Blom P. W. M. & de Leeuw D. M. Low-voltage polymer field-effect transistors for nonvolatile memories. Appl. Phys. Lett. 87, 203509 (2005).

[b32] Asadi K., Blom P. W. M., de Leeuw D. M., Conductance switching in organic ferroelectric field-effect transistors. Appl. Phys. Lett. 99, 053306 (2011).

[b33] Black C. T., Farrell C. & Licata T. J. Suppression of ferroelectric polarization by an adjustable depolarization field. Appl. Phys. Lett. 71, 2041–2043 (1997).

